# Gastro-Hysteropexy as a Safe and Reliable Means of Correcting Prolapsus and Retro-Displacement of the Uterus

**Published:** 1894-06

**Authors:** Young H. Bond

**Affiliations:** St. Louis, Mo., Professor of Diseases of Women, Marion-Sims College of Medicine, Gynecologist to Rebekah Hospital, Grand Avenue Free Dispensary, etc.


For Texas Medical Journal.

### GASTRO-HYSTEROPEXY AS A SAFE AND RELIABLE MEANS OF CORRECTING PROLAPSUS AND RETRO-DISPLACEMENTS OF THE UTERUS.

BY YOUNG H. BOND, M. D., ST. LOUIS, MO.,

Professor of Diseases of Women, Marion-Sims College of Medicine, Gynecologist to Rebekah Hospital, Grand Avenue Free Dispensary, etc.

BY FAR the most frequent displacements to which the uterus is liable are the downward and backward, viz.: the various degrees of prolapsus, retroversion and retroflexion. But few, if any, troubles in the entire field of gynecology impose more of suffering and misery than is entailed by the inflammatory consequences of these affections. The reflex disturbances and the general ill-health that usually follow, often lead to melancholia, hysteria, and even more pronounced insanity. In many instances the physical health is wrecked, and the functions of the various systems of the body are so perverted as to lead to a condition of general impairment of nutrition which predisposes to the activity of any inherited tendency to disease.

I should like to emphasize the significance of the phrase "inflammatory consequences" in this connection, for it is to these that symptoms are chiefly due; for example, pelvic peritonitis with adhesions, binding the uterus, ovaries, fallopian tubes and sometimes the bladder in unnatural positions; ovaritis and salpingitis terminating in cystic or pus formations; endometritis, cystitis, chronic constipation, etc. I am aware that these conditions sometimes predispose to, or bring about the displacements in question, but I am satisfied that in very many instances they stand in the relation of cause rather than of effect; and I am convinced from ante-mortem observations that the generally accepted idea that pelvic peritonitis, with its train of evils, is almost uniformly due to salpingitis incident to endometritis, is an error; that in reality its most frequent cause, apart from post-partum sepsis, is the pressure and consequent disturbed circulation, friction, etc., of the retroposed uterus.

I shall not presume, before this intelligent audience, to make anything like a systematic and complete presentation of the history, etiology, symptoms, physical signs and treatment of these affections; such an exposition would be appropriate before a class of students or adapted to the ends of a text-book. The purpose that I have in view in presenting this paper is to emphasize a pathological and etiological condition shared by all of these affections in their initial departure, from which proceeds a common and major principle of treatment. However, the fact should not be lost sight of that the individual affections call for special means in addition to the principle of treatment common to them all.

In order that I may make myself understood, it will be necessary that I recall to your attention the fact that in the erect posture the pelvis is placed so obliquely with reference to the trunk that the axis of its inlet is represented by a line drawn from the umbilicus to the middle of the coccyx. Now, as you know, the axis of the uterus corresponds with that of the pelvic inlet; hence, the normal position of the uterus is such that the force of intra-abdominal pressure, which is perpendicular, falls upon its posterior surface, and operates not only from above but from behind. Temporary departures from this relation of the position of the uterus and the direction of intra-abdominal pressure are constantly occurring, but they are only temporary, and therefore physiological.

Any permanent deviation from their normal relation becomes at once the prominent determining etiological factor in the production of either a prolapsus, a retroversion or a retroflexion, and the particular displacement is determined by the condition or state of integrity possessed by the suspensory supports, namely:

the long, the broad and the utero-sacral ligaments, together with the restraining supports or pelvic floor.

Now it can be appreciated that however prominent a part may be played by a breach in the pelvic floor, or by impaired suspensory supports, in predisposing to and determining the character of the displacement, yet the salient fact remains that they are but contributory agencies in their accomplishment; they determine the direction of retreat of the uterus from the cause operating above, viz.: intra-abdominal pressure. For example, if of the suspensory supports the round ligaments be alone principally elongated, and the walls of the uterus not wanting in tonicity, we will have retroversion. If to a similar state of the suspensory supports be superadded a flabby condition of the uterine walls, then we will have retroversion plus retroflexion. If the round, the broad and the utero-sacral ligaments be elongated and the integrity of the pelvic floor is wanting, we will have prolapsus.

It will be perceived that under normal circumstances the force of intra-abdominal pressure falling upon the posterior surface of the uterus, acts as an agent in maintaining the proper position of the uterus by forcing it in a direction in which descent is physically impossible; but under other circumstances it becomes the essential displacing factor. The round ligaments might be elongated, but if in spite of this circumstance the uterus did not become retroposed, so as to permit other than its posterior surface to receive the force of intra-abdominal pressure, we should not have retroversion. If the walls of the uterus were ever so flabby, they would not bend appreciably, were it not for intra-abdominal pressure. Were it not for the same pressure, lacerations of the pelvic floor would not result in loss of resiliency of the suspensory supports and consequent prolapsus.

Then the keystone in the arch of all these affections is found to be the same, viz., altered relation of the force of intra-abdominal pressure and that uterine surface to which it should be applied. The first step in prolapsus, as respects the altered position of the uterus, is a movement of the fundus backwards, and the same is true of retroversion and of retroflexion, so that it must be apparent that defects in the uterine supports operate indirectly in accomplishing the displacements in question and directly in determining their character. They permit a force which, under normal circumstances, serves to maintain the uterus in its proper position, to become the chief factor in the production of its displacement.

In dealing with aggravated cases of distortions, such as fall within the purpose of this paper, we have very largely lost sight of this, the central idea that should engage us in our methods of treatment. For, however we may seek to redress imperfections of the supports, and repair lacerations of the pelvic floor, if we fall short of placing the uterus in such a position as respects intra-abdominal pressure as to cause it to cease to be an element in the production of displacement, we utterly fail to obtain satisfactory and enduring results; since it is only a question of time when the gathered-up tissues of a colporrhaphy, perineorrhaphy, etc., will have yielded to the steadily continued downward intra-abdominal pressure. Experience has amply proven this observation, and the explanation of the fact is found in the circumstance that I have sought to make clear.

DIAGNOSIS.

To the end that treatment of these affections may be successful, it is a matter of prime and paramount importance that we should be able to make a reliable diagnosis. The gynecologist that is in the habit of proving his diagnosis through laparatomy work, if he uses the proper means and methods of physical exploration of the pelvic organs, will soon become so proficient that he can safely venture upon a positive diagnosis in fully 95 per cent of his cases.

By method and means of investigation, I mean, that the patient having been thoroughly anæsthetized, must be placed in the dorsal position, with the limbs well flexed upon the body, so as to cause the sacral curve to be brought well to the front, to the end that the axis of the pelvic cavity will conform as nearly as possible to that of the trunk. By this means the space between the fingers of the right hand that are in the vagina and rectum, and those of the left hand that are buried through the abdominal muscles above the symphisis, will be materially lessened, so that each organ of the pelvis can be clearly outlined, and its form, position, consistency, mobility, etc., readily determined. I usually employ the index finger in the vagina, and the middle finger in the rectum simultaneously. The greater amount of information is obtained through the rectum.

TREATMENT.

The retroversion pessary finds its useful application in those cases of retroversion and retroflexion in which the uterus is not bound down by peritoneal adhesions; in which the ovaries are not prolapsed, and as is often the case, incarcerated in the cul-de-sac of Douglas; and in those in which the displacement has not lasted so long that the round ligaments and other supports have lost all power of regaining their resiliency. In such cases, having first relieved complicating conditions, such as laceration of the cervix, tears of the perineum, etc., we can by the patient and judicious use of the intra-vaginal or Smith-Hodge pessary accomplish much for the relief of our patients, and recognizing the fact that the displacements usually manifest themselves after parturition, it becomes the imperative duty of the obstetrician, to make a careful physical exploration of the pelvic organs within eight or ten weeks after accouchment, with a view to correct, in the manner indicated, any altered position of the uterus that may be discovered. For, at that period the displacement, as a rule, has not produced inflammatory results which would negative the use of the pessary; since such cases generally remain uncomplicated for a variable period of time, often months, save possibly by the results of subinvolution.

The condition and results of treatment are far different when the displaced organ or associated pathological state has produced pelvic peritonitis, the adhesive products of which have fastened the uterus, and possibly the ovaries, in varying relations to each other in the cul-de-sac of Douglas, with possibly suppurative results: so also are they different when the suspensory supports, from long continued traction, have become so attenuated as to be practically paralyzed. Just as we are able to destroy the tendency of rubber to contract by overstretching, so likewise do the suspensory supports lose their function.

Prolapsus in the first degree can usually be corrected by the use of the pessary, after complicating conditions have been relieved, such as hypertrophy, elongation or lacerations of the cervix, or tears of the pelvic floor; but let the case beecome once well established, as is represented in the remaining degrees of prolapsus, and we have a condition of things represented by stretched paralyzed suspensory supports; the cellular tissue is without elasticity, the muscles of the pelvic floor, if not torn, are atrophied, and often have undergone fatty degeneration. Under such circumstances, with the conditions recounted, it is idle, nay more, it is foolish, to expect anything like complete and satisfactory results from the methods of treatment usually pursued.

In the case of the adherent retroposed uterus, pessaries are inapplicable, and forcible breaking up of the adhesions, after the method of Schultz, through the uterine cavity, is dangerous and often ineffectual; in fact, all attempts to replace or otherwise interfere with a uterus that is bound down in its displaced position by adhesions, other than through intra-abdominal procedure, is dangerous, and to be condemned, for the reason that absolutely correct knowledge of the pathological conditions cannot always be ascertained, and in consequence we may unconsciously compromise the life of a patient by causing the contents of a pus sac to be liberated into the peritoneal cavity; and further, it is a blind procedure, lacking all the elements of precision necessary to reliable and good ends. Here it is that a method of treatment presents itself which in the hands of the clean and skilled surgeon is both safe and efficient. I allude to hysteropexy, which, as you know, means literally a fixing of the uterus, and is applied to those procedures that have for their purpose the maintenance of the fundus of the uterus in connection with the anterior abdominal wall.

The methods in practicing hysteropexy pursued by operators thus far, differ in many particulars; the operation has been practiced scarcely long enough to enable us to decide positively, which is the most desirable method, and yet long enough to permit us to pronounce quite confidently upon its worth. Through it, in a sure and reliable manner, we place the uterus so that its posterior surface is opposed to intra-abdominal pressure, which, as previously stated, acts from above and behind in such a manner as to assist in maintaining the proper position of the uterus. We thus supply the keystone to the arch of uterine retension, without which, in no uniformly reliable manner, can the damaged pillars of support be made adequate to their requirements. It is astonishing how small a measure of force is necessary to keep the uterus anteposed when anchored ever so slenderly in its proper place.

HYSTEROPEXY.—The general precautions pertaining to a cœliotomy having been observed, the opening through the abdominal wall is made as low down as practicable, and no larger than necessary to admit of efficient and expeditious work. The patient is placed in Trendelenberg's position, by means of which the pelvis is freed of the confusing presence of intestines and omentum, and they are spared the injury of unnecessary manipulation. The walls of the incision are held outward and apart to admit of as free inspection of the pelvic organs as possible. Parenthetically I will say, that the advantages in this direction are very great in subjects with thin abdominal walls, and the reverse if heavily loaded with adipose tissue. Our manipulations in the abdomen are also facilitated or retarded by these conditions. By means of inspection and the sense of touch accurate knowledge of the conditions and relations of the pelvic structures is obtained. If adhesions exist, they are broken up by means of the index and middle fingers; the ovaries and fallopian tubes are brought up to the abdominal opening and carefully inspected, and if found to be seriously diseased they are removed, otherwise not. Small ovarian cysts are treated by clipping off a portion of the cyst wall. The fact that the organs are found adherent is not in itself, sufficient justification for their removal. Next, the uterus having been lifted to the front, it is seized through its fundus with a double tenaculum and held by an assistant in such relation to the abdominal wound that the operator can readily pass a curved needle threaded with a heavy chromicised catgut suture through all the tissues of abdominal wall, except the skin, embracing sufficient of them to secure a firm hold, then through the anterior and upper portions of the fundus, and out similarly through the abdominal wall at the opposite side of the incision. The tenaculum is now removed, and the assistant takes the catgut suture in its stead. The abdominal wound is then closed by interrupted suture in the usual manner, with this difference: that the catgut suture that has transfixed the uterus is tied before, but not until the abdominal suture in closest relation to it has been drawn upon, so as to approximate the peritoneal surfaces. The tying of this last abdominal suture draws the skin over the catgut suture and thus buries it. This has been my method of operation, and the results have been uniformly good. In not a single instance has the uterus failed to remain in the position in which it was placed, and the outcome, so far as the restoration of the general health is concerned, has been all that could be expected, and in many instances signally excellent. It has been the custom of some operators to abrade the anterior surface of the uterus, with the view of producing extensive peritoneal adhesions; of others to transfix the uterus with many sutures, and pass the same through all the structures of the abdominal wall, using as a rule silk-worm gut. I think that a larger experience will demonstrate that such extra precautions are unnecessary.

It may be asked by some one, why not do Alexander's operation for the relief of these affections? The fact of the matter is that Alexander's operation has a very limited field of utility; it is entirely inapplicable to cases of adherent uteri, or where the adnexa are diseased. It will accomplish scarcely more than a properly adjusted pessary when the round ligaments have not become permanently paralyzed; it will accomplish nothing when they have become so, for they are then merely greatly attenuated chords, often very difficult to find. The element of danger as respects hysteropexy is great, or almost nil, according as the operator is wanting in proper antiseptic precautions and pathological knowledge or the reverse. In the one case the patients usually die very promptly, in the other they are scarcely conscious of discomfort after the expiration of the twenty-four hours following the operation.

The query may come to you, what will be the result in the event of pregnancy following hysteropexy? Our experience in this respect has been limited, but not altogether untoward. I believe that the use of a suture such as catgut, that undergoes absorption, will be conducive to good results in this direction.

REPORTS OF CASES.

During the year just passed very few weeks have elapsed that I have not practiced hysteropexy in one or more cases, in connection usually with other surgical procedures, for such cases as call for this operation are usually complicated. So far I have had no deaths, and as yet the uterus remains as placed in every case. It is true that the length of time that has passed is not very great, yet it is more than sufficient for the institution of the initial steps of displacement, the scheme of which, together with the results to other operators, justify a sense of assurance as to the ultimate outcome. I will not tax your patience by impressing upon you numerous and extended reports of cases. I shall merely ask your attention to such as exemplify a principle of treatment.

The first case that I will report is that of Mrs. F. age 32 years, married, the mother of four children; ill health dates from a confinement five years previous to the time of consulting me in the early part of September, 1893. Her general health was horribly impaired: weight 105 pounds; suffering more or less all the time; utterly incapacitated for any duty. Physical exploration revealed an irregular mass in the cul-de-sac. Diagnosis: retroflexed and abherent uterus, in connection with prolapsed adherent and suppurating ovaries. Operation on 27th of September, 1893. Adhesions broken up with great difficulty. Material assistance to this end was rendered by Dr. B. M. Hypes, who elevated the parts by pushing with his fingers in the posterior fornix of the vagina. Ovaries and tubes removed and the uterus fastened to the anterior abdominal wall after the manner previously described. Patient made a good and prompt recovery. Weight now 137 pounds, ruddy complexion, magnificent spirits. Examination of the uterus made a short time since finds it in normal position, all pelvic induration gone, and seemingly the usual degree of mobility of the parts.

Case 2. As illustrative of the conservative possibilities from hysteropexy, I report the following:

Mrs. G., aged 27, married, the mother of three children, consulted me on February 1st, 1894; general health greatly impaired, dating from last confinement two years previous. Diagnosis: Uterus retroverted and bound down by adhesions; ovaries cystic, prolapsed, and adherent. Operation on February 19, 1894. Adhesions broken up, one ovary removed, uterus approximated to the anterior abdominal wall as before. Prompt recovery. Uterus in proper position at the present time. General health completely restored.

Case 3. Multiple operations at one sitting. Mrs. L., aged 31, married; one child; ill health dates from its birth, about three years ago. General health greatly impaired; suffering constantly with pain in the back, down the limbs, and a sense of downward pressure in the pelvis. Diagnosis: Laceration of the perineum, laceration of the cervix, prolapsus of the uterus and cystic ovaries. Chloroformed March 12, 1894, at which time was done a double trachelorraphy, a perineorraphy, also one ovary was removed, the other treated by snipping off a portion of the cyst walls, and the uterus approximated to the abdominal wall in the usual manner. Length of time under the anæsthetic less than one hour. Her recovery has been uninterrupted. The uterus at the present writing remains as adjusted.

Case 4. Mrs. W., aged 38, mother of four children, fleshy; complains of constant pain in the region of the sacrum, very nervous and frequently melancholic. Diagnosis: Retroflexed and adherent uterus, ovaries propalsed and adherent in the cul-de-sac under the uterus. Operation February 23, 1894. Adhesions broken up, no structures removed, and uterus approximated as in other cases. Result, recovery. A recent examina tion of the patient reveals the parts in good condition and mobility measurably restored. The ovaries did not prolapse after the uterus was brought to the front.

Case 5. Mrs. R., aged 39, mother of three children, consulted me in January, 1893; general health completely wrecked. Her features and expression were those of an old woman shrivelled by age; mental faculties so disturbed as to make her almost irresponsible most of the time; decidedly melancholic. Diagnosis: Retroflexed and adherent uterus, prolapsed and suppurating ovaries, also adherent; pachy-salpingitis. Operation February 12, 1893. Adhesions broken up, diseased structures removed, and uterus fastened to the abdominal wall after the manner practiced by me. Result, complete recovery; general health, mentally and bodily, thoroughly restored. One who had seen the patient just prior to the operation would scarcely recognize her to-day as being the same person, for she is now the picture of health, having gained fully forty pounds of flesh. The uterus still remains in proper position.

It is my custom to curette the uterus almost invariably before performing hysteropexy, for the reason that endometritis is generally an accompaniment of chronic displacement.

